# Feasibility of a new bariatric fully endoscopic duodenal-jejunal bypass: a pilot study in adult obese pigs

**DOI:** 10.1038/s41598-022-24614-7

**Published:** 2022-11-24

**Authors:** Jean-Michel Gonzalez, Sohaib Ouazzani, Stephane Berdah, Nicolas Cauche, Cecilia Delattre, Joyce A. Peetermans, Agostina Santoro-Schulte, Ornela Gjata, Marc Barthet

**Affiliations:** 1 Department of Hepatogastroenterology, Faculty of Medicine, Aix-Marseille University, Chemin des Bourrely, 13915 Marseille, Cedex 20, France; 2 Department of Gastroenterology, Hôpital Erasme, Brussels, Belgium; 3 Department of Digestive Surgery, Hôpital Nord, Assistance Publique-Hôpitaux de Marseille, Aix-Marseille University, Marseille, France; 4 Centre for Surgical Teaching and Research (CERC), Aix-Marseille University, Marseille, France; 5 Brussels Medical Device Center (BMDC), Brussels, Belgium; 6 Endoscopy Division, Boston Scientific Corporation, Marlborough, MA USA

**Keywords:** Gastroenterology, Obesity

## Abstract

This study assessed a new natural orifice transluminal endoscopic surgery (NOTES) bariatric endoscopic procedure in obese adult pigs. This 14-week prospective study compared 6 adult male Yucatan test pigs with baseline mean age 1.5 years to 2 control pigs. The test pigs received a fully endoscopic NOTES-based duodenal-jejunal bypass including measurement of the bypassed limb and creation of a gastrojejunal anastomosis (GJA) using a gastrojejunal lumen-apposing metal stent (GJ-LAMS) at Week 0, placement of a duodenal exclusion device (DED) at Week 2, and endoscopic examinations at Weeks 6 and 10. At Week 14, the pigs were sacrificed for necropsy. All endoscopic procedures were technically successful. By Week 14, the controls had gained a mean 1.1 ± 2.1 kg (+ 1.6%) while the intervention animals lost a mean 6.8 ± 3.9 kg (− 10.5%) since baseline. GJ-LAMS migrations occurred in 3 pigs, two of which also had DED migration and/or partial dislocation. Two other pigs died, one with aberrant splenic vein positioning near the GJA and the other from an unknown cause. An endoscopic bariatric bypass procedure with controlled bypass length was technically successful in all the cases and led to weight loss in test animals. Safety concerns must be further addressed.

## Introduction

Since 1985, endoscopic bariatric treatments have been developed as minimally invasive techniques and/or devices using flexible endoscope access, mainly for weight loss as well as resolution of associated comorbidities^1^. In 2015, a joint task force convened by the American Society for Gastrointestinal Endoscopy and the American Society for Metabolic and Bariatric Surgery defined acceptable efficacy and safety thresholds, proposing that endoscopic bariatric treatment intended as a primary obesity intervention in individuals with Class II/III obesity (body mass index > 35 kg/m^2^) should achieve a mean minimum threshold of 25% excess weight loss measured at 12 months, that any nonprimary endoscopic bariatric treatment (e.g., early intervention, bridging, or metabolic therapy) should achieve 5% of total body weight lost as the absolute mean minimum threshold, and that an acceptable associated risk level is ≤ 5% incidence of serious adverse events^2^. Intragastric balloons^2^ and endoscopic suture gastroplasty^3^ have met these criteria. Some other procedural endoscopic bariatric treatments have been slower to demonstrate a high level of efficacy with a low rate of associated adverse events.

Since 2020, our research team has tested a natural orifice transluminal endoscopic surgery (NOTES) gastrojejunostomy in animal studies as a potential alternative^4,5^ or bridge^6^ to bariatric surgery. This procedure uses short-term placement of a gastrojejunal lumen-apposing metal stent (GJ-LAMS) to create a gastrojejunal anastomosis (GJA) with a controlled bypass length and a dedicated duodenal exclusion device (DED) to induce weight loss. Our studies to date suggest that the baseline endoscopic procedure^5^ and conversion to a one-anastomosis duodenal-jejunal bypass^6^ may be feasible in juvenile pigs. A similar animal study published in 2021 used endoscopic sleeve gastroplasty for gastric volume reduction followed by a GJA and pyloric closure, without dedicated devices or postprocedural longitudinal follow-up^7^. Durability of the GJA and weight loss after these procedures have not been tested in an adult obese animal model.

We present a pilot study of the NOTES endoscopic gastrojejunostomy procedure in obese adult pigs. Stability of GJA patency and device migration patterns were examined.

## Methods

### Prospective fully endoscopic procedure and survival animal study

**Figure 1:**
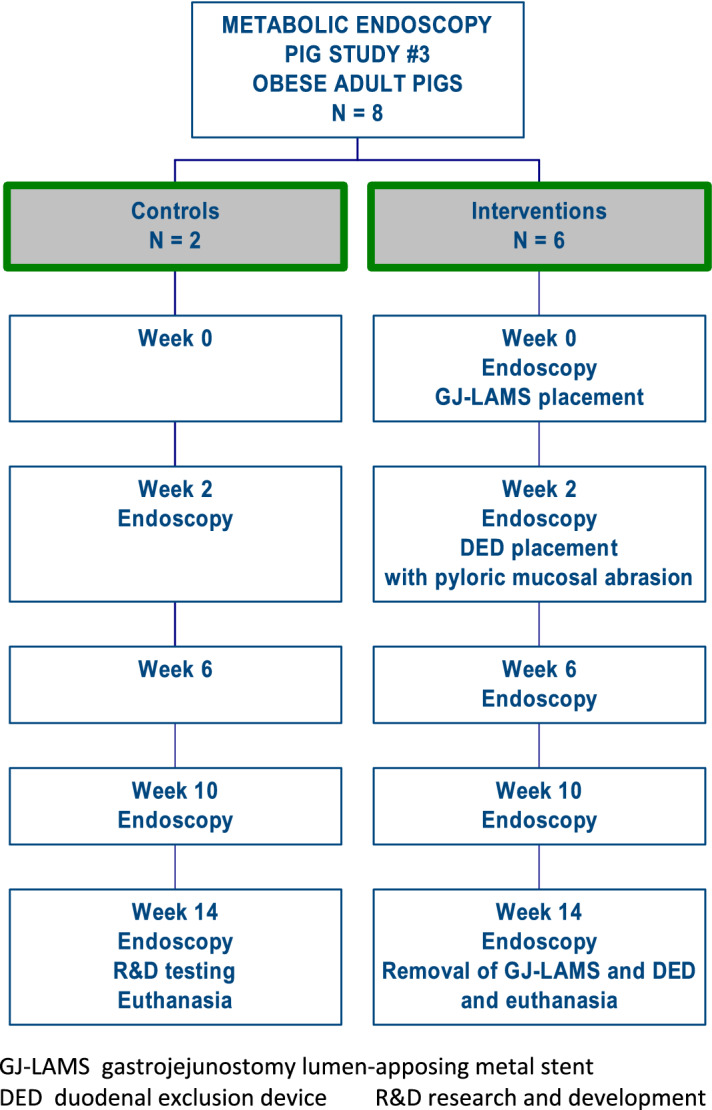
Study flow chart. GJ-LAMS gastrojejunostomy lumen-apposing metal stent, DED duodenal exclusion device, R&D research and development.

A 14-week prospective animal study was conducted between May 19 and August 25, 2021 by physician procedural experts at an academic animal research facility (Centre for Surgical Teaching and Research, Marseille, France) (Fig. 1). The Ministere de L'enseignement Superieur, de La Recherche et de L'innovation (Ministry of Higher Education, Research and Innovation, Paris, France) granted ethics approval for the study. All applicable institutional and/or national guidelines for the care and use of animals were followed under approval of the Institutional Animal Care and Use Committee (IACUC) and the ethical principles of the Canadian Council of Animal Care (CCAC). The study animals were obese adult Yucatan minipigs acquired from the Institut National de la Recherche Agronomique (INRA, Rennes, France). INRA induced obesity in the minipigs before their acquisition for the study. Because of their adult age at baseline, potential future weight change was expected to be influenced by the study interventions (including study diet and endoscopic procedures), not by growth. Animals were maintained on a commercial high-protein piglet diet (Bermond P203-GN-Porcelet) consisting of 2.3% crude fat, 4.2% crude fiber, and 16.5% crude protein.

Animal handling, preprocedural care and anesthesia were handled as reported previously^5,6^. The study was reported in accordance with the ARRIVE guidelines^8^.

### Endoscopic procedures

The procedure of the NOTES endoscopic duodenal-jejunal bypass with controlled limb length procedure was performed as reported previously by our team^5^. Four individual devices were used for the fully endoscopic jejunal bypass procedure: Enteral light Beacon, Atraumatic Grasper, a modified 20 mm dedicated lumen-apposing metal stent (GJ-LAMS), and Duodenal Exclusion Device (DED) (Boston Scientific Corporation, Marlborough, Massachusetts, USA, and Brussels Medical Device Center, Brussels, Belgium). The procedure involved: (1) at week 0, endoscopic measurement of the bypassed limb by jejunal insertion of a dedicated light beacon (150-cm target length), followed by NOTES-based creation of a GJA using a GJ-LAMS, (2) at week 2, pyloric occlusion with a dedicated duodenal exclusion device (DED).

The DED underwent modifications since our previous studies^5,6^. The current design was modified to an asymmetric dumbbell to enhance pullout force while maintaining tissue contact with uncoated regions. Endoscopic mucosal resection (EMR) was done on the gastric side of the pyloric ring using a hot snare and was associated to electrocautery abrasion if required in order to treat the whole pyloric circumference. DED uncoated regions and EMR were used to promote tissue ingrowth for decreasing the migration rate. The GJ-LAMS device was the same design as in previous studies^5,6^.

### Monitoring and follow-up

Oral intake, animal wellbeing and adverse events were monitored daily per protocol and animal facility standard procedures. The interventional pigs were fasted for one day before every endoscopic procedure, and on the day of the procedure. On post-procedure day 1, they were given water with sucrose (after GJ-LAMS placement) or water (after DED placement). Subsequently, they received a slurry containing water mixed with the following amounts of the high-protein piglet feed: 250 g on post-procedure days 2 and 3, 500 g on post-procedure day 4, and 750 g on post-procedure day 5, then 1 kg per day of dry piglet feed for the remainder of the follow-up period. The control pigs were given the same dietary regimen on the same schedule as the interventional pigs.

Pigs’ weights were recorded at each endoscopic intervention while the animals were anesthetized for procedures, namely on Weeks 0 (placement of GJ-LAMS), 2 (placement of DED), 6, 10, and 14 (final endoscopy and euthanasia and necropsy). If additional endoscopic examinations occurred (e.g. in response to concerning signs or symptoms), weight was also recorded at those times.

### Final endoscopic examination

Per our earlier published protocol^5^, a final endoscopic examination was conducted on each pig at Week 14, specifically observing for gastric stasis; inflammation, ulceration and adherence; pylorus mucosal appearance and opening, and duodenal inflammation, stenosis or patency.

### Euthanasia and necropsy examination

#### General examination

At 14 weeks of follow-up after GJ-LAMS placement, euthanasia was performed using pentobarbital 3 g per animal while the animals were under general anesthesia. In case of death before this time, postmortem endoscopic examination was performed to evaluate whether devices were in place and assess the appearance of adjacent tissue.

As in our previous study^5^, necropsy was performed including gross examination of the peritoneal cavity to look for signs of a prior leak, inflammation, abscess or adhesions; of the stomach and small bowel to look for inflammation or distention; and of the GJA and pylorus to look for inflammation, induration or adhesions. Histopathological examination was performed in all surviving pigs at the end of the follow-up. The bypassed limb length was measured and jejunal tissue was examined for distension or strictures.

#### Examination of GJA patency after GJ-LAMS removal or migration

GJA patency was examined visually at the time of each endoscopic evaluation, and the diameter was measured at the end of the study during necropsy on Week 14 in most surviving pigs.

### Endpoints

Endpoints were (1) technical success (including placement and removal of GJ-LAMS and DED), (2) animal weight change from baseline, (3) symptom change, (4) device-related or procedure-related adverse events (AEs), and (5) gross and microscopic examination findings at necropsy.

### Statistical analysis

Descriptive statistics were tabulated for continuous variables (weight, duration of procedures). Serial weights were plotted for each animal at the time of each endoscopic examination, and the change in weight between baseline and Week 14 was calculated. Observed weights were plotted versus corresponding weights from the control pigs. Statistical analyses were performed using SAS software, version 9.4 (SAS Institute Inc., Cary, NC, USA; https://www.sas.com).

## Results

### Baseline characteristics

All 6 interventional animals were male Yucatan pigs, with a mean age of 534 days (1 year, 5.5 months) at baseline. The 2 control animals were male Yucatan pigs, both aged 608 days (1 year, 8 months) at baseline.

### Technical success

**Figure 2:**
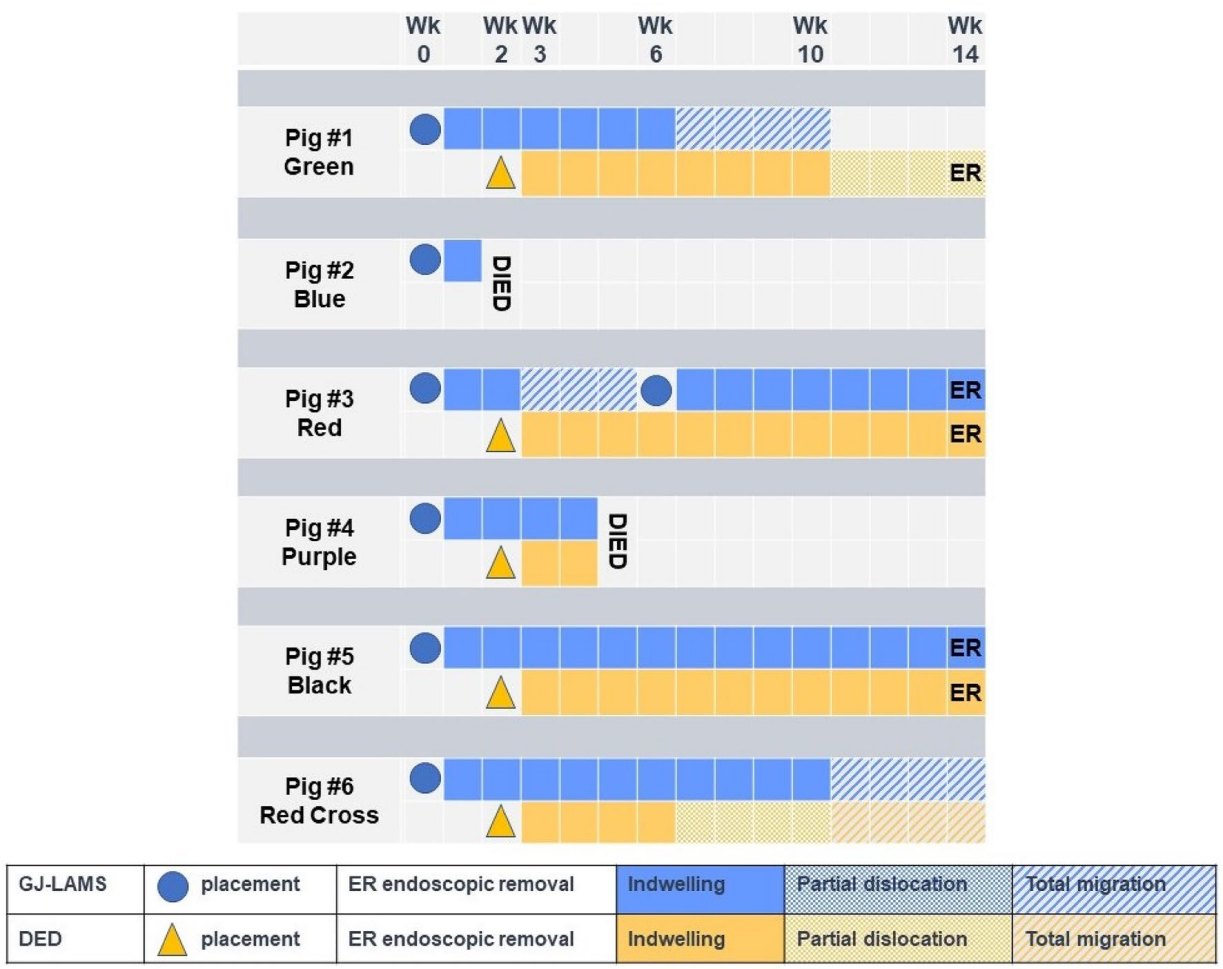
Placement, removal and migration of GJ-LAMS and DED in adult domestic pigs.

The depth of beacon in the jejunum reported during the procedure was estimated to be 135 cm in Pig Black and Pig Red Cross and 155 cm in the other 4 pigs. Some difficulty advancing the light beacon (rated 3 or 4 on a scale of 1 [easiest] to 10) was documented for Pig Green, Pig Black and Pig Red Cross. Placement of the GJ-LAMS was technically successful in all 6 interventional pigs (Video S1), and removal was successful in the 2 surviving pigs without a GJ-LAMS migration (Pig Red, Pig Black) (Fig. 2). All GJ-LAMS were placed in the body of the stomach, with a mean placement time of 41.7 ± 7.1 (range 32.0–50.0) minutes.

DED placement was completed successfully in all 5 interventional pigs that survived 2 weeks after GJ-LAMS placement, with a mean placement time of 9.6 ± 0.9 (range 9.0–11.0) minutes (Video S2). Some difficulty with DED placement (rated 3 on a scale of 1 [easiest] to 10) was documented for Pig Green and Pig Red Cross.

### Animal weight change from baseline

**Figure 3:**
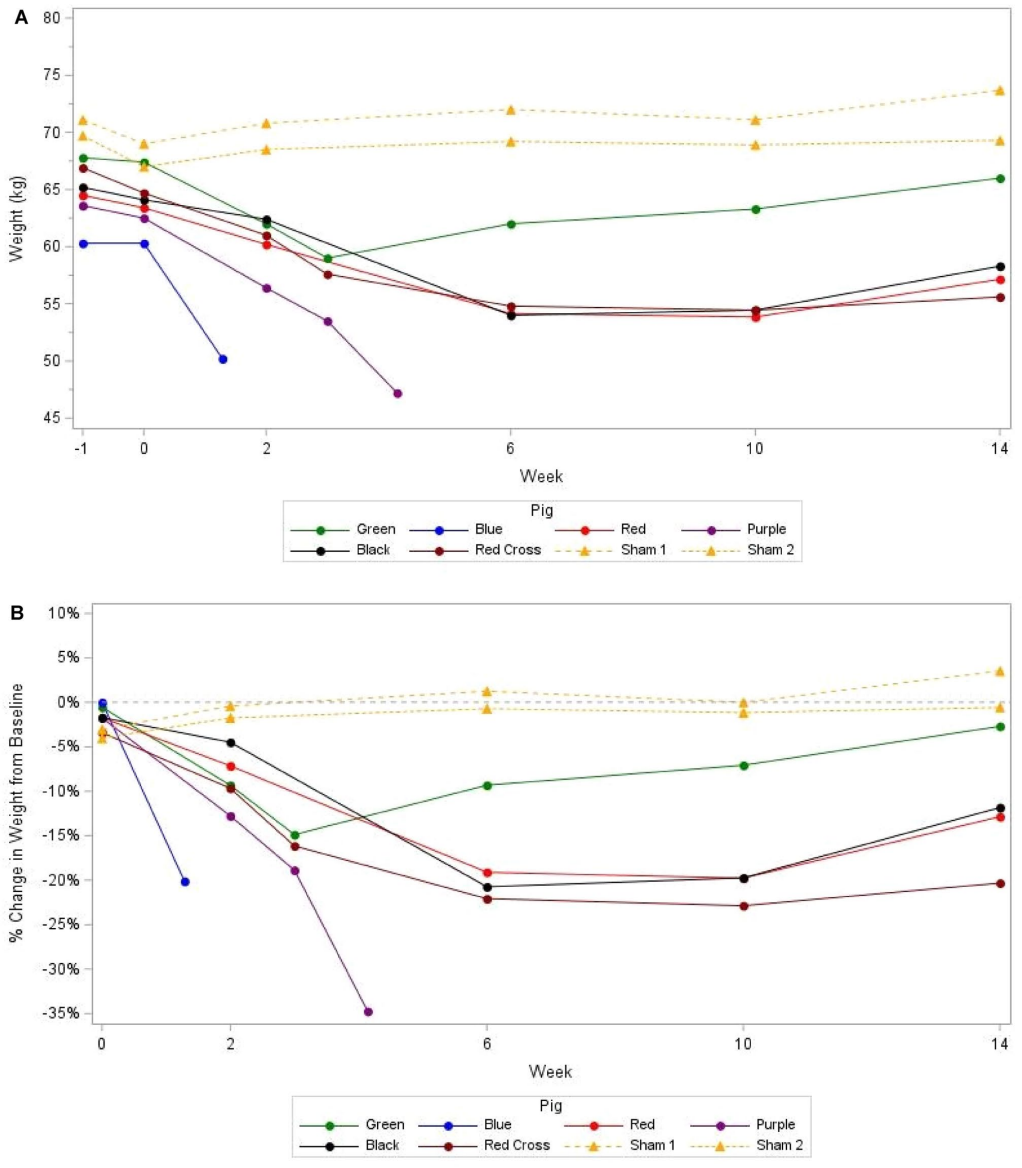
Absolute (**A**) and relative (**B**) weight change from Week 0 to Week 14.

The preprocedural baseline weights (before Week 0) were 70.4 ± 1.0 kg for the controls and 64.7 ± 2.7 kg for the intervention animals. At Week 0, both groups had lost weight, but the control animals still had higher mean body weight than the intervention animals (68.0 ± 1.4 kg vs. 63.7 ± 2.4 kg respectively) (Fig. 3A).

The controls showed modest weight gain during follow-up while the intervention animals lost weight. By Week 14, the control animals had 71.5 ± 3.1 kg mean body weight with mean 1.1 ± 2.1 kg gained (1.6% mean gain vs. baseline/preprocedural), while the intervention animals had mean 59.3 ± 4.6 kg body weight at week 14 with mean 6.8 ± 3.9 kg lost (10.5% mean weight loss vs. baseline/preprocedural) (Fig. 3B).

Pig Green lost less weight than the other intervention pigs. Pig Green reached a weight nadir at Week 3, after which it gained weight at each subsequent examination but did not exceed its baseline weight by Week 14 (Fig. 3A). Of note, Pig Green was found to have a migrated GJ-LAMS at Week 10 (since Week 6) (Fig. 2). The GJ-LAMS was not replaced. At the Week 14 necropsy examination, Pig Green was found to have a stenotic GJA (10 mm diameter), a 618-cm-long limb, and a partially dislocated DED.

### Change in symptoms

At Week 3, Pig Green, Pig Red Cross and Pig Purple stopped eating for 2 days despite normal water-drinking and behavior. An extra endoscopic examination at Week 3 showed that the GJ-LAMS and DED were in place in all 3 animals. No reason for the reduced food intake was identified. The animals resumed normal consumption after the 2 days of symptoms.

### Device-related adverse events

#### Device migrations and partial dislocations

Asymptomatic GJ-LAMS migrations were noted in three animals during scheduled endoscopic assessment at follow-up (Fig. 2). Pig Green was observed to have absence of the GJ-LAMS at Week 10 (last observed in place at Week 6) and a stenotic GJA; no intervention was performed on the stenosis at this time. Pig Red was observed to have absence of the GJ-LAMS at Week 6 (last observed in place at Week 2); the GJ-LAMS was replaced at this time. Pig Red Cross was observed to have absence of the GJ-LAMS at Week 14 (last observed in place at Week 10), but the GJA had good patency at necropsy.

A partial dislocation of the DED was observed in Pig Green at Week 14 (last observed in place at Week 10). A partial dislocation of the DED was observed in Pig Red Cross at Week 10 (last observed in place at Week 6). At Week 14, Pig Red Cross’ DED was absent. The partial dislocations of the DED were never associated with any mucosal injury.

In summary, 3 GJ-LAMS migrated without clinical consequences. 2 partial dislocation of DED occurred and also one DED migration. All GJ-LAMS and DED in place were removed without any difficulty.

### Deaths

**Figure 4:**
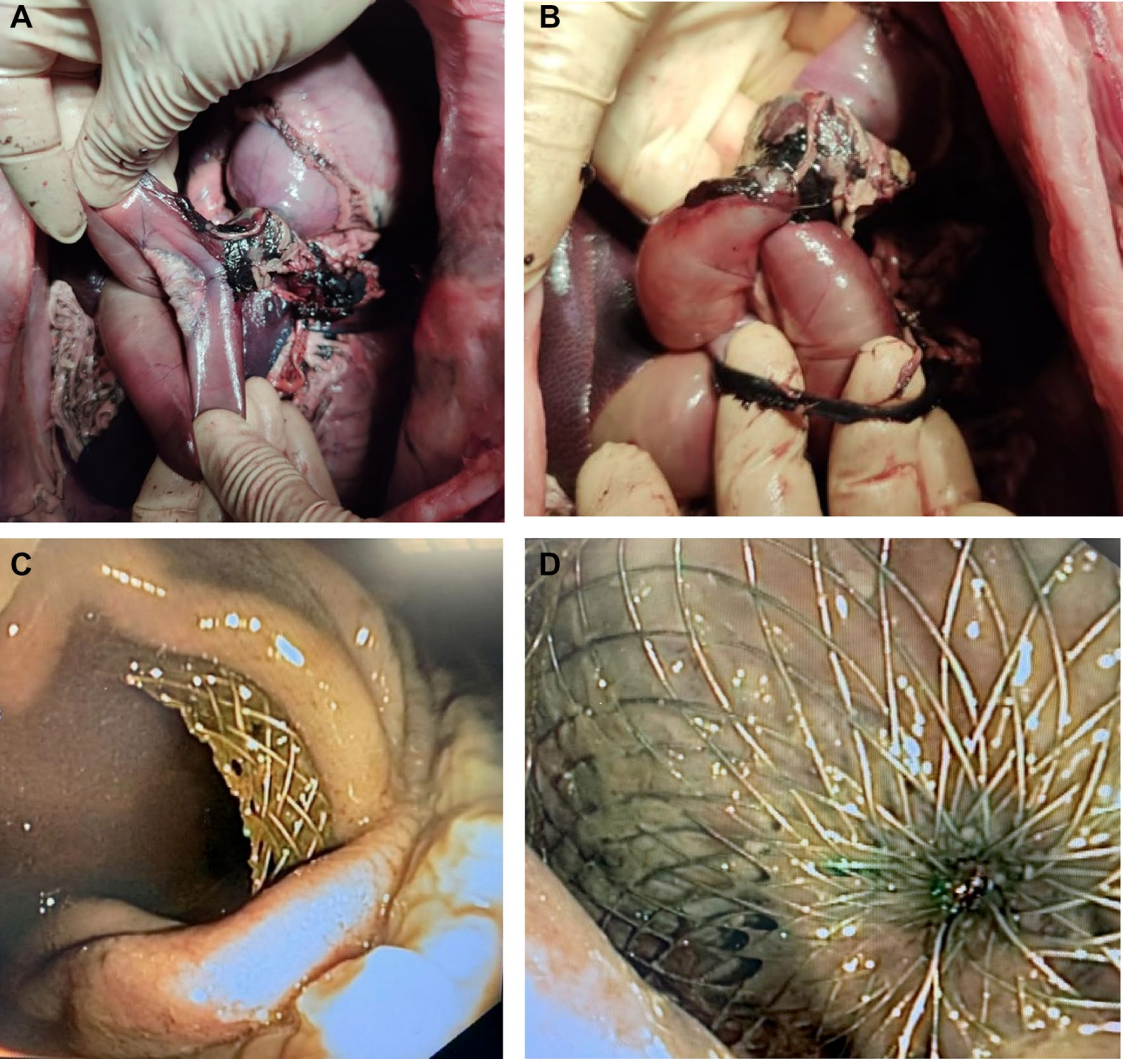
Necropsy specimens from two pigs that died. Pig Blue died 9 days after GJ-LAMS placement. Necropsy revealed the ribbon-like splenic vein strangulating the efferent limb just below the GJA, leading to intestinal obstruction (**A**, **B**). Pig Purple died from an unknown cause on Day 29 (15 days after DED placement). GJA was in place with normal patency (**C**). DED was in place with partial tissue ingrowth (**D**).

Two deaths occurred in interventional animals during follow-up. On Day 9 (9 days after GJ-LAMS placement), Pig Blue was found dead. Necropsy showed gastric liquid stasis with the splenic vein wrapped around the efferent loop at the base of the GJA, suggesting dysfunction of the GJA leading to intestinal obstruction (Fig. 4A–B). No adherence, inflammation or abscess was seen.

On Day 29 (15 days after DED placement), Pig Purple was found dead. Endoscopic examination showed that the GJA was in place with normal patency and the DED was in place (Fig. 4C–D). No reason for the death for was identified.

### Necropsy examination and histopathological findings

#### General examination

**Figure 5:**
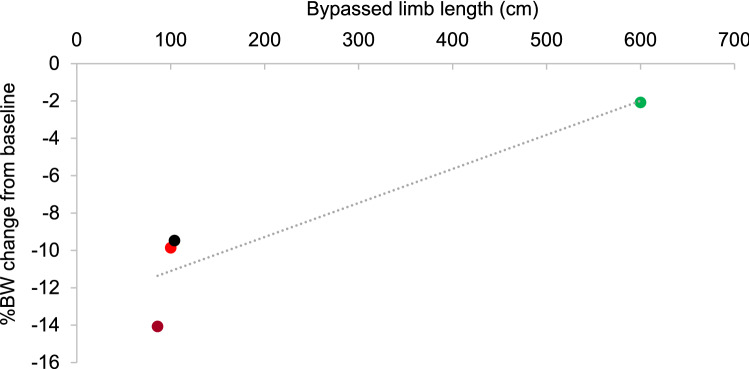
Bypassed limb length as a function of percent body weight change in interventional pigs.

At necropsy, no inflammation, no peritonitis, no visible abscess or adhesion were found in any of the pigs. Mean duodenal-jejunal bypass limb length upon necropsy examination 226.3 ± 261.3 cm (range 86.0, 618.0) among the 4 surviving pigs. Despite experiencing little weight loss, Pig Green had an exceptionally long limb length of 618 cm (Fig. 5) related to difficult insertion of the light beacon, while the other pigs had limbs ranging from 86 to 104 cm.

#### GJA and pyloric patency after device removal or migration

The GJ-LAMS used in this study created a GJA with a diameter of 20 mm. Pig Red and Pig Purple had their GJ-LAMS removed at the time of the final endoscopic assessment immediately before necropsy, and their GJA stomas were therefore fully patent. Pig Green, which had its GJ-LAMS absent for a minimum of 4 weeks or maximum of 8 weeks, exhibited a GJA diameter of 10 mm (50% reduction from its initial size). In Pig Red Cross, the GJA was observed to have good patency after GJ-LAMS absence of ≤ 4 weeks, although the diameter size was not recorded. The size of the pyloric sphincter was not measured, but moderate pyloric stenosis was reported in 2 (Pig Red and Pig Black) out of the 3 pigs undergoing DED removal. Regardless of DED status, none of the animals showed signs of inflammation, perforation, or ulceration in the pyloroduodenal area.

#### Histopathological examination

**Figure 6:**
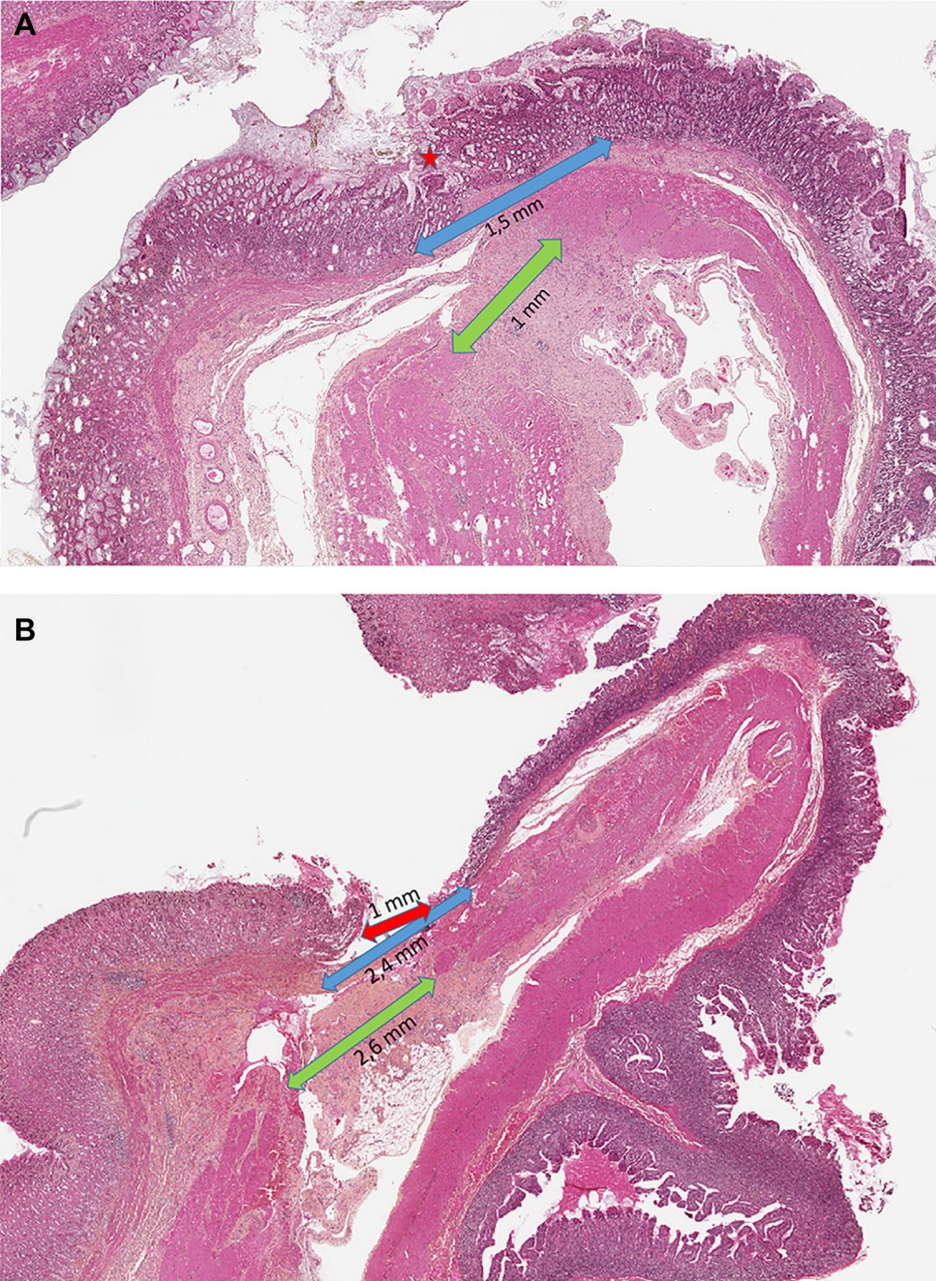
(**A**) Histopathological examination at the gastrojejunal anastomosis of Pig Green: incomplete fusion of anastomotic wall. Stars denote complete fusion of and arrows reflect fibrotic distance between intestinal tissue layers: mucosae (red); 1.5-mm and 1-mm fibrotic area interposed at the junction of the muscularis mucosa layer (blue) and muscularis layer (green), respectively. (**B**) In Pig Red Cross, mucosal ulceration (1 mm) (red arrow); 2.4-mm and 2.6-mm fibrotic area interposed at the junction of the anastomotic muscularis mucosa layer (blue arrow) and the muscularis layer (green arrow), respectively.

All surviving pigs received histopathological examination at the end of follow-up (Table 1). Complete parietal fusions were associated with a short fibrotic interspace ranging from 1 to 2.6 mm (Fig. 6A and B). No leak, fistula or any dehiscence was seen. No parietal abscess, or perianastomatic or peritoneal inflammation was evident. Liver biopsy samples from all surviving pigs were normal, without evidence of steatosis.

**Table 1:** Histopathological findings at the gastrojejunal anastomosis.

Histological finding	Black	Green	Red	Red cross
Mucosa layer fusion	Yes	Yes	No	No
Superficial ulceration (diameter)	No	No	Yes (5 mm)	Yes (1 mm)
Parietal abscess	No	No	No	No
Muscularis mucosa layer fusion	No	No	No	No
Muscularis externa fusion (fibrotic distance)	No (1.8 mm)	No (1 mm)	No (2 mm)	No (2.6 mm)
Peritoneal inflammation	No	No	No	No

## Discussion

This 14-week pilot study in an adult porcine model showed that a fully endoscopic NOTES-based bypass procedure for bariatric indications was technically successful and was associated with weight loss in intervention pigs compared to controls. However, two of 6 intervention pigs died and device migrations and GJA stenosis occurred making long-term efficacy uncertain.

Metabolic/bariatric surgery is currently the most effective treatment for obesity, providing profound, sustained weight loss^9^ and improvement or resolution of obesity-related comorbidities^10,11^. Bariatric surgery has been demonstrated to be substantially more effective than intensive lifestyle intervention at inducing sustained weight loss and diabetes remission^12,13^. However, less than 1% of eligible patients in the US undergo bariatric surgery every year^14^. Endoscopic techniques could potentially broaden availability of bariatric interventions to more patient populations, but efficacy and safety have only begun to be demonstrated. Procedures that are simple enough for a large number of endoscopists to perform have not been developed.

Our fully endoscopic metabolic procedure has been tested in two studies of juvenile pigs and the current study of adult Yucatan pigs. The current study was the first to suggest efficacy to induce weight loss compared to controls over 3.5 months of follow up. While these results are encouraging, continued device-associated adverse events cause safety concerns that warrant further preclinical studies for device and procedural development. Based on the findings, we will take the following actions in future studies:

1. Considering the anomalous event related to the splenic vein in deceased Pig Blue, we will use a peritoneal approach on the animal’s median or right side (not left side).
2. Device migrations continued to occur in the over half of the intervention animals. Device and procedural modifications will continue to be tested to decrease the rate of migration.
3. GJA stenosis occurred in Pig Green. We will continue to study approaches to control the GJA diameter, preferably without the need for extra endoscopic examinations.
4. The exceptionally long bypass limb in Pig Green was attributed to difficulty with the light beacon. Some human studies suggest that longer bypassed limb lengths are associated with greater weight loss^15^, which is inconsistent with Pig Green’s minimal amount of weight loss despite a very long limb. Based on studies supporting that a 100–200 cm combined length of biliopancreatic and alimentary limb gives optimum results with Roux-en-Y gastric bypass in most patients^16^, we will continue to aim for a total limb length in this range in future studies.

Our study had strengths and limitations. Unlike earlier pilot studies in growing animals^4,5^, this study used an obese adult animal that allowed better simulation of the human target population. We used a control group to more accurately judge the effect of the intervention. Our study was limited by a small sample size and deaths in two of six interventional animals. Also, stenosis of the GJA occurred in one of the 4 surviving animals, and continued device migrations occurred; to achieve adequate safety, these issues need to be resolved in future studies. Because GJA patency measurements were only performed in pigs with non-migrated GJ-LAMS and in one of two pigs with found to have a GJ-LAMS migration at the time of necropsy, we cannot accurately estimate the long-term impact of LAMS removal or migration on GJA narrowing.

In conclusion, NOTES-based endoscopic bypass procedure was technically successful in an adult porcine animal model but led to serious adverse events and device migrations. Two of six pigs died, one for unknown reasons. The described procedures are pioneering and still in the early developmental stage, so attempts by less experienced endoscopists are not recommended. Improved safety (especially lack of deaths) must be demonstrated before additional studies are performed. Our team is working to add safety measures, one being performing the light beacon progression and creation of the gastrojejunal anastomosis under X-ray guidance in live pigs. Optimization of GJA diameter and reduction in device migrations are also necessary before clinical testing can be considered. In future studies, we hope to add gastric volume reduction as described in another published endoscopic bypass procedure^7^.

## Supplementary Information

Supplementary Information 1.

Supplementary Video 1.

Supplementary Video 2.

## Data Availability

The data that support the findings of this study are available from Boston Scientific Corporation but restrictions apply to the availability of these data, which were used under license for the current study, and so are not publicly available. Data are however available from Professor Marc Barthet (Marc.BARTHET@ap-hm.fr) upon reasonable request and with permission of Boston Scientific Corporation.
