# Supplementary material for: Feasibility of a new bariatric fully endoscopic duodenal-jejunal bypass: a pilot study in adult obese pigs
Source: Sci Rep. 2022 Nov 24;12:20275. doi: 10.1038/s41598-022-24614-7 (PMC9700790; doi:10.1038/s41598-022-24614-7)
Supplement: Supplementary file 1 — Supplementary Information 1. [file 41598_2022_24614_MOESM1_ESM.docx]

**Video S1. Deployment of gastrojejunal lumen-apposing metal stent (GJ-LAMS) to form a gastrojejunal anastomosis.**

**Video S2. Deployment of duodenal exclusion device (DED) to occlude the pylorus.**
